# Draft Genome Sequence of *Rhodococcus* sp. Strain 66b

**DOI:** 10.1128/genomeA.00229-17

**Published:** 2017-05-25

**Authors:** Louise F. Thatcher, Cindy A. Myers, Cathryn A. O’Sullivan, Margaret M. Roper

**Affiliations:** CSIRO Agriculture and Food, Centre for Environment and Life Sciences, Wembley, Western Australia, Australia

## Abstract

We report here the draft genome sequence and annotation of *Rhodococcus* sp. strain 66b isolated from the soil of southwest Western Australia. This strain exhibits a range of bioactivities, including plant growth promotion, biosurfactant production, and wax degradation. Whole-genome sequencing was conducted to uncover the underlying mechanisms.

## GENOME ANNOUNCEMENT

Within the *Actinobacteria* phylum, species from the genus* Rhodococcus* are significant for their bioremediation and industrial applications, for example, the production of enzymes or metabolites involved in degradation of organic compounds or the production of biosurfactants or bioflocculants (1). We isolated and purified *Rhodococcus* sp. strain 66b from naturally water-repellent soils based on its wax-degrading ability and further assessed it for biosurfactant production based on surface tension assays (2). The production of biosurfactants was associated with its potential to degrade waxes that cause water repellency in sandy soils. Sequencing of 16S rRNA designated strain 66b a *Rhodococcus* species (2, 3).

DNA for whole-genome sequencing was extracted from mycelia and coccoid/rod-shaped fragments using a Mo Bio PowerLyzer UltraClean microbial DNA isolation kit, followed by preparation of an indexed Illumina TruSeq library (350-bp insert) and sequencing using 150-bp paired-end reads on an Illumina MiSeq instrument by the Australian Genome Research Facility (AGRF), Melbourne, Australia. A total of 0.48 Gbp of raw data were generated from a sequence run using approximately 1/10 of a sequencing lane. Reads were assessed for quality, trimmed [CutAdapt (4)], and sorted as per Thatcher et al. (5), and overlapping reads were merged using FLASH (version 1.2.11) (6). *De novo* assembly of reads (paired-end, singletons, merged) was performed using SPAdes (version 3.9.0) (7) with the “--careful” option and k-mer lengths of 21, 33, 55, and 77. Contigs less than 1,000 bp were removed. The 66b genome was assembled into 6.68 Mbp (57 scaffolds; *N*_50_ count, 7 scaffolds; N50 length, 0.38 Mb), with a G+C content of 62.3%. Coding sequences, functional annotation, and secondary metabolite biosynthesis gene clusters were predicted by Prokka (version 1.11) (8) [incorporating Prodigal (version 2.6.3) (9)], Blast2GO (version 1.0.2) (10), and antiSMASH (version 3.0.5.1) (11), respectively.

Blast2GO (10) best BLAST hits analysis for species comparisons revealed the closest neighbor strain for 66b to be *Rhodococcus erythropolis*. Genome annotation by Prokka (8) allowed for the identification of 6,359 coding sequences and 54 tRNAs within the 66b genome. Prediction of secondary metabolite clusters by antiSMASH (11) suggested the 66b genome harbors at least 18 biosynthetic gene clusters, of which 7 are nonribosomal peptide synthetases. Other biosynthetic gene clusters include 2 polyketide synthases and a bacteriocin, ectoine, terpene, and butyrolactone cluster, suggesting its potential to produce metabolites contributing to its bioactivities and potential to improve soil wettability.

### Accession number(s).

This whole-genome shotgun project has been deposited at DDBJ/ENA/GenBank under the accession no. MUZB00000000. The version described in this paper is version MUZB01000000.
